# Whipple’s Disease Mimicking Sarcoidosis

**DOI:** 10.7759/cureus.41839

**Published:** 2023-07-13

**Authors:** Pawan KC, Frehiywot K Ayele, Sabin Karki, Madeeha S Waleed

**Affiliations:** 1 Rheumatology, Emory University School of Medicine, Atlanta, USA; 2 Internal Medicine, Suburban Community Hospital (Lower Bucks Hospital), Bristol, USA

**Keywords:** immunosuppression, pas stain, non-caseating granuloma, sarcoidosis, whipple's disease

## Abstract

Whipple's disease is a rare systemic disease caused by a *Tropheryma whipplei *infection. Although older literature reports a low rate of incidence, case reports continue to rise due to increased awareness of the disease. Classic Whipple's disease presents as weight loss, diarrhea, and arthralgia and may involve the heart, central nervous system (CNS), or any other organ system. Some patients with Whipple's disease do not have the classic signs and symptoms of the disease.

We present a case of Whipple's disease in a patient with poor appetite, weight loss, and granulomatous inflammation of various organs, including the kidneys and spleen, mimicking sarcoidosis. She had presented three years earlier with acute kidney injury (AKI) and hypercalcemia. The renal biopsy revealed diffuse granulomatous interstitial nephritis. Both AKI and hypercalcemia resolved with prednisone; however, her weight loss and decreased appetite continued. The initial positron emission tomography (PET) scan showed increased fluorodeoxyglucose (FDG) avidity in the spleen and large intestine, and the splenic biopsy revealed non-caseating granulomas. A diagnosis of sarcoidosis was made, and she was started on methotrexate with prednisone.

Nevertheless, the weight loss and poor appetite were relentless. A repeat PET scan showed increased FDG avidity in loops of the small and large intestines. A small intestinal biopsy revealed positive periodic acid-Schiff (PAS) and negative acid-fast bacilli (AFB) revealing the diagnosis of Whipple's disease. Whipple's disease should be considered in the differential diagnosis of sarcoidosis, especially in those patients worsening on standard immunosuppression.

## Introduction

Sarcoidosis is a granulomatous disorder of multiple organs, with the lungs and lymphatic systems being the most frequently affected sites of the body [1]. Since sarcoidosis is a diagnosis of exclusion, it requires ruling out all other possible diagnoses. On immunological and histopathological examination, it reveals non-caseating granulomas [2]. It is often misdiagnosed; a tissue biopsy is needed for a definitive diagnosis [3]. Although the typical finding is non-caseating granuloma, it is not pathognomonic of sarcoidosis as it can be seen in a variety of infectious, non-infectious, and neoplastic conditions. In a study of 308 biopsies with non-caseating granulomas, sarcoidosis was the leading cause of non-caseating granulomas, but in 44% of cases, there was an alternate diagnosis. It was estimated that more than a quarter of the initial diagnoses will be changed based on biopsy results and clinical course [4]. Sarcoidosis is a rare mimic of Whipple's disease [5]. We present a case of Whipple's disease, which was initially misdiagnosed as sarcoidosis.

## Case presentation

A 45-year-old female presented to the rheumatology clinic with poor appetite and weight loss for the past five years. She was admitted to an outside hospital three years prior to presentation for similar complaints, where she was found to have acute kidney injury (AKI) and hypercalcemia. Her creatinine was elevated to 2.6 mg/dL (0.6-1.2) from a baseline of 0.7 mg/dL, calcium was elevated at 12.1 mg/dl ( 8.6-10.3), and parathyroid hormone (PTH) level was appropriately suppressed at 3.7pg/ml (15-65 ). Serum phosphorus level was 3.7mg/dl (3.4-4.5), and alkaline phosphatase was 90IU/L (44-147). Serum protein electrophoresis (SPEP) showed a polyclonal increase in gamma globulin, and urine protein electrophoresis (UPEP) showed a band of restricted mobility possibly representing a monoclonal protein; however, immunofixation was negative. Her 1,25-dihydroxyvitamin D and serum angiotensin-converting enzyme (ACE) levels were within the upper limits of normal. Her 25-hydroxyvitamin D and parathyroid hormone-related peptide (PTHrp) levels were normal. The 24-hour urine protein showed sub-nephrotic proteinuria. The skeletal survey for multiple myeloma was negative. A CT scan of the chest without contrast showed mildly enlarged mediastinal lymph nodes bilaterally but no other significant abnormalities. 

She was treated with intravenous hydration, with which her calcium and creatinine improved to 9.7 mg/dL and 2.2 mg/dL, respectively. A renal biopsy demonstrated diffuse acute granulomatous interstitial nephritis. Hence, she was started on prednisone 40 mg daily for suspected sarcoidosis. Her kidney function normalized, following which the prednisone was tapered down to 5 mg over a period of one year. The patient declined the initiation of a steroid-sparing agent.

In the following months, the patient developed intermittent epigastric abdominal pain along with decreased appetite and continuous weight loss. Attempts were made to wean her off the steroids, however, her symptoms worsened with the attempted taper. Lab investigations in subsequent visits showed leukocytosis, thrombocytosis, and a low mean corpuscular volume (MCV). Further workup by hematology showed an elevated C-reactive protein (CRP) of 71.8 mg/L (<10), iron saturation of 5%, and ferritin of 89 ng/ml (11-307). Peripheral blood flow cytometry was negative for the monoclonal cell population. The patient was then referred to rheumatology for further management of presumed sarcoidosis. A positron emission tomography (PET) scan was obtained to assess sarcoidosis activity and evaluate potential alternate diagnoses. The PET scan revealed intense, diffuse uptake in the cecum and proximal colon. Subsequently, she underwent a colonoscopy with a biopsy of the colonic mucosa, both of which were unremarkable. The discrepancy between the abnormal PET and the normal colonoscopy led to the concern that prednisone might be masking the complete findings. Therefore, a repeat PET scan was obtained after weaning the patient off prednisone. It revealed an interval decrease in the previously noted intense avidity of the cecum, with a small residual focus of uptake. Multiple prominent, hypermetabolic retroperitoneal lymph nodes were seen, and there was a diffuse splenic uptake. A splenic biopsy demonstrated a non-necrotizing granuloma that was negative for acid-fast bacilli (AFB) and Grocott methenamine silver (GMS) stain. A bone marrow biopsy was negative for granulomas or other marrow infiltrative processes. Considering sarcoidosis as the most likely explanation, the patient was restarted on prednisone with the addition of methotrexate as a steroid-sparing agent. The patient then relocated to a different state and established care with a new rheumatologist. 

**Figure 1:**
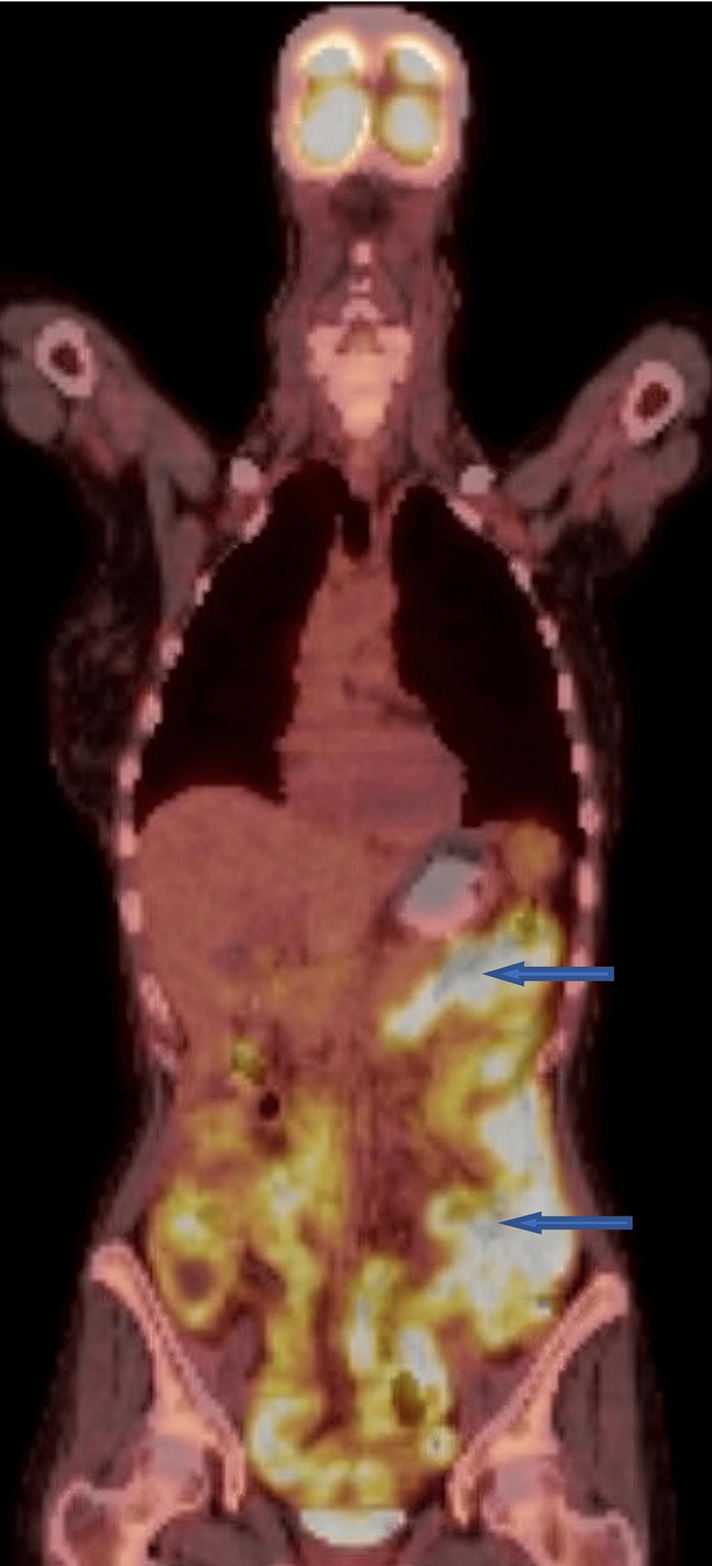
The FDG PET scan reveals diffuse intense FDG avidity (blue arrows) throughout much of the small and large bowel FDG: Fluorodeoxyglucose, PET: Positron emission tomography

Shortly after relocating, she began having a poor appetite, early satiety, weight loss, and fatigue, requiring hospitalization. Gastric emptying studies were normal, and an MRI abdomen showed nonspecific fluid-filled dilated bowel loops. She was discharged following improvement with symptomatic treatment. However, following the discharge, her symptoms recurred. A new whole-body PET scan was obtained, which showed intense fluorodeoxyglucose (FDG) activity throughout the loops of the small and large bowels (Figure 1). This finding was considered highly atypical for sarcoidosis as there was no extraintestinal activity. 

**Figure 2:**
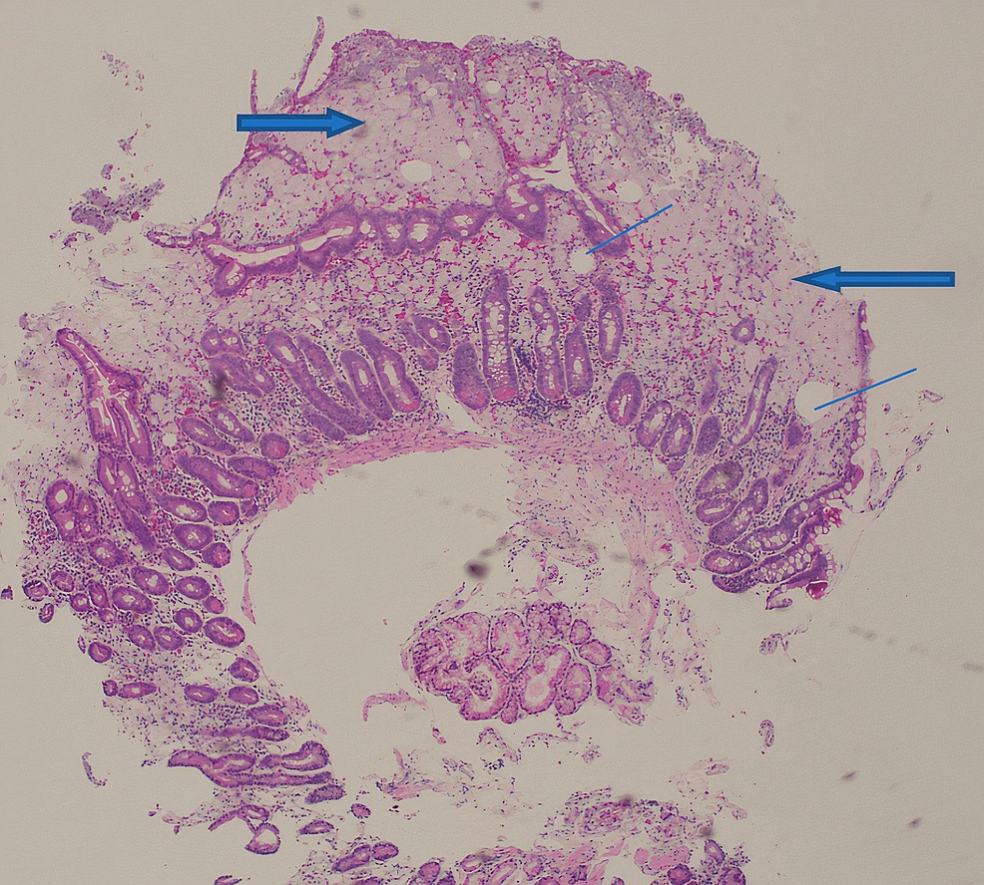
The duodenal mucosa shows extensive involvement of lamina propria by foamy macrophages (bold blue arrows) and lipoid vacuoles (blue lines)

Therefore, esophagogastroduodenoscopy (EGD) with push enteroscopy to the third portion of the duodenum was performed, which showed multiple patchy areas of friable and granular mucosa with contact bleeding. Surgical biopsies revealed numerous foamy histiocytes distending the blunted villi with lipoid vacuoles (Figure 2).

**Figure 3:**
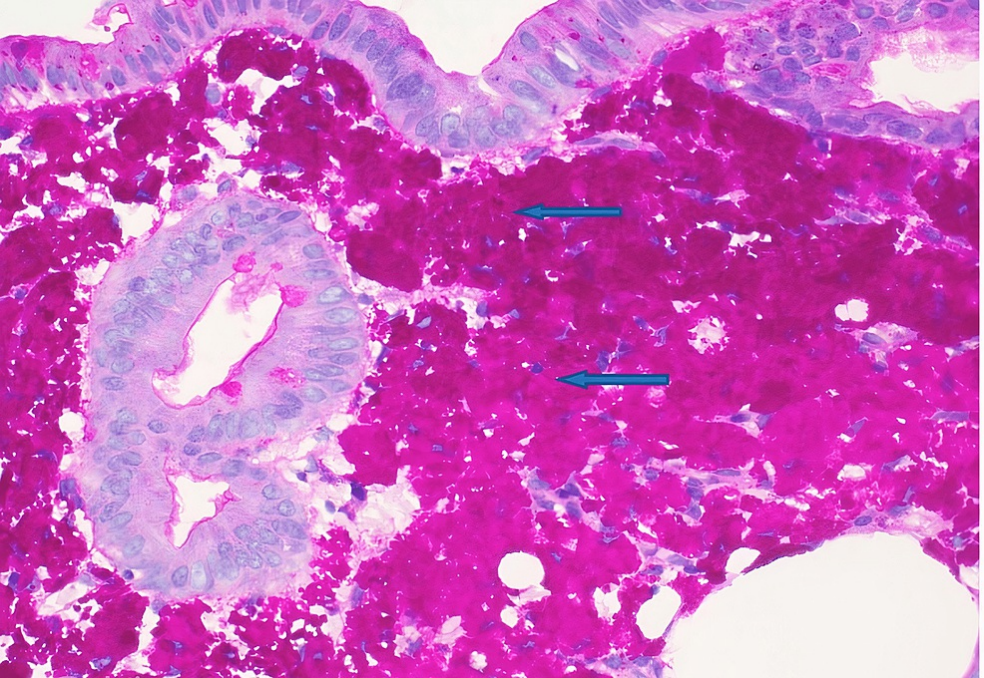
Macrophages containing intensely red granular material (blue arrows) of Tropheryma whipplei

The macrophages were periodic acid-Schiff (PAS) positive and AFB negative, confirming the diagnosis of Whipple's disease (Figure 3). 

Consequently, methotrexate was discontinued and prednisone was tapered off. The patient was initiated on IV ceftriaxone 2 gm daily for 14 days, followed by oral trimethoprim-sulfamethoxazole 160 mg to 800 mg twice daily for 12 months per infectious disease recommendations. On subsequent clinic visits, she was noted to have a progressive improvement in appetite and weight. Diarrhea and abdominal pain had subsided. Approximately 11 months after the treatment, she reported an excellent appetite and had gained 19 kg. She is now at her baseline weight of 59.5 kg from 40.5 kg before the diagnosis.

## Discussion

Whipple's disease has an insidious course and is more common in males than females, with a ratio of 2-3:1 [6]. There are only a few studies that have examined its incidence and prevalence. Some studies suggest a prevalence of 3/1,000,000 and an incidence of 0.1-0.6/1,000,000 new cases in Western populations. Some estimates have been as low as 12 new cases per year worldwide [7]. 

Whipple's disease can be described as a systemic bacterial infection caused by *Tropheryma whipplei*, with diarrhea, weight loss, abdominal pain, and arthralgia as the most common though not invariable manifestations [8]. It typically involves the duodenum and the small intestine [9]. Half of the patients have lymphadenopathy, especially mesenteric and mediastinal lymph nodes [10].

In this case, the presence of granulomas on renal and splenic biopsies originally led to the diagnosis of sarcoidosis. Although the patient recovered briefly on immunosuppression, the ensuing months were marked by frequent relapses of digestive symptoms. Isolated digestive tract involvement (digestive tract sarcoidosis (DTS)) is extremely rare in sarcoidosis, described in 0.1% to 1.6% of cases. Very few cases of small intestine sarcoidosis have been reported, and post-mortem involvement in one series of 320 autopsies was only 0.03%. Patients with DTS often have multi-visceral lesions. Sarcoidosis more often involves the superior digestive tract, especially the stomach. The absence of ileal or colonic involvement has been independently associated with DTS [11,12]. Investigations resulting in findings atypical for sarcoidosis on the repeat PET scan in this case ultimately led to the diagnosis of Whipple's disease.

A large cohort of more than 1200 patients with pulmonary sarcoidosis found that kidney manifestations were present in 12% of cases, although the disease may have been silent or undetected for many years [13]. Renal involvement has been hypothesized as a late feature of Whipple's disease; however, it was the initial presenting feature in our case, with later worsening of systemic symptoms [14]. The presence of bacteria demonstrated by electron microscopy and bacterial antigen demonstrated by immunofluorescence within the kidney have been described. 

This case brings into question the possibility of the co-existence of sarcoidosis and Whipple's disease. However, this would be an extremely rare occurrence. On the basis of 20,000 autopsies performed at the Institute of Pathology of Basel, it was calculated that this chance association of sarcoidosis and Whipple's disease coexistence may happen once in 108autopsies [15]. This favors the assumption that in our case, the renal involvement was due to Whipple's disease. The patient's striking clinical response to antibiotics and lack of worsening upon withdrawal of immunosuppression strongly support this conclusion.

Our patient was initially diagnosed with granulomatous disease of the extraintestinal organs. In extraintestinal Whipple's disease, the density of *T. whipplei* is often so low that routine histological stains for microbes, including PAS stain, are negative [16]. Notably, in our patient, the PAS stain was negative on the splenic biopsy. Splenic granuloma in Whipple's disease has not been described in the literature to our knowledge. Therefore, Whipple's disease should be included in the differential diagnosis of granulomatous diseases such as sarcoidosis, especially in someone who is not responding to typical immunosuppression. 

Multiple studies have shown that diarrhea in Whipple's disease can be triggered by medical immunosuppression. Thus, patients have been shown to develop digestive symptoms such as diarrhea after starting treatment with corticosteroids, methotrexate, and azathioprine. Several publications also report on immunosuppression and the exacerbation of Whipple's disease [17]. In our case, the patient developed worsening abdominal pain and diarrhea following the initiation of steroids and methotrexate. It is quite possible that immunosuppression accelerated the appearance of these digestive symptoms.

## Conclusions

Whipple's disease is a mimic of sarcoidosis and should be considered in multi-system granulomatous disease that is not responding to standard immunosuppression. Immunosuppression can either unmask or exacerbate the underlying Whipple's disease. Antibiotics are the mainstay of treatment for Whipple's disease, in contrast to immunosuppressive therapy for sarcoidosis. 
